# Synthesis, Characterization, and Acute Oral Toxicity Evaluation of pH-Sensitive Hydrogel Based on MPEG, Poly(**ε**-caprolactone), and Itaconic Acid

**DOI:** 10.1155/2013/239838

**Published:** 2013-11-30

**Authors:** Liwei Tan, Xu Xu, Jia Song, Feng Luo, Zhiyong Qian

**Affiliations:** State Key Laboratory of Biotherapy and Cancer Center, West China Hospital, West China Medical School, Sichuan University, Chengdu 610041, China

## Abstract

A kind of chemically cross-linked pH-sensitive hydrogels based on methoxyl poly(ethylene glycol)-poly(caprolactone)-acryloyl chloride (MPEG-PCL-AC, PECA), poly(ethylene glycol) methyl ether methacrylate (MPEGMA, MEG), N,N-methylenebisacrylamide (BIS), and itaconic acid (IA) were prepared without using any organic solvent by heat-initiated free radical method. The obtained macromonomers and hydrogels were characterized by ^1^H NMR and FT-IR, respectively. Morphology study of hydrogels was also investigated in this paper, and it showed that the hydrogels had good pH-sensitivity. The acute toxicity test and histopathological study were conducted in BALB/c mice. The results indicated that the maximum tolerance dose of the hydrogel was higher than 10000 mg/kg body weight. No morality or signs of toxicity were observed during the whole 7-day observation period. Compared to the control groups, there were no important adverse effects in the variables of hematology routine test and serum chemistry analysis both in male or female treatment group. Histopathological study also did not show any significant lesions, including heart, liver, lung, spleen, kidney, stomach, intestine, and testis. All the results demonstrated that this hydrogel was nontoxic after gavage. Thus, the hydrogel might be the biocompatible potential candidate for oral drug delivery system.

## 1. Introduction

Hydrogels are networks which can swell in water and hold a large mount of water while maintaining their three-dimensional structure. Over the past decades, the environment-sensitive hydrogels which are also called “intelligent” or “smart” hydrogels have attracted increasing attention, because they can show a sudden or gradual change in their dynamic and equilibrium properties with the changes of ambient conditions such as temperature [1, 2], pH [3–5], and ionic strength [6]. They might have great potential in targeted drug delivery system, on-off switches for modulated drug delivery, artificial organs, and immobilization of enzyme due to their biocompatibility and resemblance to biological tissues [7–11]. Among the various types, pH-sensitive hydrogels based on biocompatible copolymers, had been widely used in the area of drug delivery system [12–18], especially oral drug delivery system [19–21]. The pH-sensitive hydrogels could adjust their swelling behavior in different pH value. In acidic gastric environment, the hydrogel is shrinking, and the drug is sustained in the hydrogel, but in high-pH intestinal tract, the hydrogel is swelling, and the drug is released from hydrogel. So the pH-sensitive hydrogels could improve drug efficacy and reduce the side effects [22–24].

Poly(ethylene glycol) (PEG) and poly(*ε*-caprolactone) (PCL) have been widely utilized as a biomaterial for their great biocompatibility. Since Perret and Skoulios [25] prepared a series of block copolymer based on PEG and PCL, this kind of copolymer have been widely researched, containing PEG, PCL, and poly(ethylene glycol) methyl ether (MPEG) that followed [26, 27]. In our previous works, we successfully prepared MPEG-PCL amphiphilic macromonomers and integrated them in the main constituent of smart pH-sensitive hydrogels [28]. The carboxyl groups (–COOH) make the hydrogel pH-sensitive. In our previous work [29], itaconic acid (IA) was chosen to prepare a new kind of pH-sensitive hydrogel due to its two carboxyl groups in one molecule, which would achieve a better pH-responsive ability. Moreover, the IA was water-soluble, and we could use water as the solvent. This would avoid the use of organic solvents considering the safety of hydrogel. The obtained hydrogels showed good pH-sensitivity and were employed to delivery protein, dexamethasone, DOX-loaded nanoparticles, and so on.

Until now, we had studied the cytotoxicity of MPEG-PCL-AC (PECA) copolymer primarily by MTT method [29]; however, the toxicological study on pH-sensitive hydrogel based on PECA copolymer has not been thoroughly examined. Since the safety evaluation of the smart hydrogel can have a significant implication on its further applications as a biocompatible carrier for oral drug delivery system, in this paper, the acute oral toxicity evaluation of pH-sensitive hydrogel based on MPEG, PCL, and itaconic acid was performed.

## 2. Materials and Methods

### 2.1. Materials

Among the whole materials, poly(ethylene glycol) methyl ether (MPEG, Mn = 2000), *ε*-caprolactone (*ε*-CL), N,N′-Methylenebisacrylamide (BIS), itaconic acid (IA), poly(ethylene glycol) methyl ether methacrylate (MPEGMA, Mn = 475), tin (II) 2-ethylhexanoate, acryloyl chloride (AC), triethylamine (TEA), and ammonium persulfate (98%) (APS) were purchased from Aldrich Company, USA. For the rest of the reagents, they were used as received. All the reagents were completely analytic grade. Male and female BALB/c mice were purchased from the Laboratory Animal Center of Sichuan University.

### 2.2. Synthesis of PECA Macromonomer and P(ECA-IA-MEG) Hydrogel

The MPEG-PCL copolymer was synthesized by ring-opening polymerization of *ε*-caprolactone initiated by MPEG using tin (II) 2-ethylhexanoate as catalyst [29, 30]. And the PECA macromonomer was obtained by dissolving MPEG-PCL in dewatered methylene chloride to react with AC, which has been reported before [29].

With APS as the initiator and BIS as the cross-linking agent, the P(ECA-IA-MEG) hydrogel was synthesized by heat-initiated free radical method. As shown in Table 1, the predetermined amounts of PECA, IA, MPEGMA, BIS, and APS were dissolved in water. Then, the entire system was soaked and bathed in water at 37°C for 4 hours. The obtained hydrogel was immersed in a plenty of distilled water for 7 days, and the water was refreshed everyday to remove the unreacted substances. The purified hydrogels were freeze-dried kept. Finally, the freeze dried hydrogels were grinded into powder and suspended in 1% aqueous sodium carboxymethyl cellulose (CMC-Na) solution for acute oral toxicity test.

### 2.3. Characterization of PECA Copolymer and P(ECA-IA-MEG) Hydrogel

#### 2.3.1. ^1^H-Nuclear Magnetic Resonance Analysis (^1^H-NMR)

The structure of PECA copolymer was analyzed by ^1^H-NMR spectrum, which was recorded on Varian 400 spectrometer (Varian, USA) at 400 MHz using deuterated chloroform (CDCl_3_) as solvent and tetramethylsilane (TMS) as internal reference standard.

#### 2.3.2. Fourier Transform Infrared (FTIR) Analysis

The chemical structures of PECA copolymer and P(ECA-IA-MEG) hydrogel were analyzed by the FTIR (KBr) spectrum, which were recorded on Nicolet 200SXV meter (Nicolet, USA). The samples were scanned at wave number range of 4000–400 cm^−1^.

#### 2.3.3. Scanning Electron Microscopy (SEM)

The morphology of the P(ECA-IA-MEG) hydrogel was recorded on JEOL SEM (JSM-5900LV, JEOL, Japan). The hydrogels were immersed in the aqueous solution of pH 1.2 and pH 6.8, respectively. And the samples were lyophilized, transected, and sputtered gold before observed.

#### 2.3.4. Swelling Behavior of the Hydrogels

The hydrogels were immersed in aqueous medium with different pH conditions (pH 1.2 and pH 7.4) at 37°C for 1, 2, 4, 8, 24, 48, and 72 h, respectively. Then they were taken out and the surplus surface water was removed by filter paper. This experiment was repeated 3 times, and the wet weights of them were recorded carefully. The swelling ratios could be calculated by the following equation:

(1) $$\begin{array}{c} \text{Swelling}\;\text{ratio}\;\left(\text{SR}\right)=\frac{W_{t}}{W_{0}}\times 100\%, \end{array}$$

where *W*_0_ was the initial dry weight and *W*_*t*_ was the wet weight of the hydrogel at time *t*, respectively.

### 2.4. Acute Toxicity Test

#### 2.4.1. Maximal Tolerance Dose (MTD)

Owing to the great biocompatibility and the swelling ability of P(ECA-IA-MEG) hydrogel, no lethal dose or median lethal dose (LD_50_) could be obtained according to our preliminary trials. So, the Maximal Tolerance Dose (MTD) method was adopted to evaluate the acute oral toxicity of the hydrogel.

All BALB/c mice were housed individually in a standard animal room maintained at a temperature of 20–22°C and a relative humidity of 50–60%. The animals which had an average weight of 20 g were 9-10 weeks old on the day of dosing. Twenty mice of both sexes were divided into two groups, hydrogel group (*n* = 10, 5 male and 5 female mice) and control group (*n* = 10, 5 male and 5 female mice). All the mice were fasted approximately 15 h prior to dosing, and water was provided continuously. Hydrogel group was given P(ECA-IA-MEG) hydrogel suspension twice at an interval of four hours by gavage, at a total dose of 10000 mg/kg body weight and a total volume of 0.4 mL/10 g body weight. Accordingly, control group was treated with a same volume of 1% CMC-Na solution. The animals were given food again approximately 4 h after dosing.

After the final administration, all the animals were continuously observed for 7 days. Observations were conducted twice daily, including morality, injury, abnormal behavior, and the general condition (the hair, activity, feces, behavior pattern, and other clinical signs). Besides, body weights for all mice were recorded on study days 1, 3 5, and 7. The necropsies of dead animals were performed to observe the gross pathological changes. On the study day 7, all the animals were sacrificed.

#### 2.4.2. Hematology Routine Test

Whole blood samples from each rat were collected by removing the eyeball for blood routine test which is determined by a hematology Analyzer (MEK-6318, Nihon Koden). And hematology routine test in our study included the following variables: white blood cell count (WBC), red blood cell count (RBC), hemoglobin (HGB), hematocrit (HCT), platelet count (PLT), mean corpuscular volume (MCV), mean corpuscular hemoglobin (MCH), mean corpuscular hemoglobin concentration (MCHC), red cell distribution width (RDW), mean platelet volumes (MPV), and platelet distribution width (PDW).

#### 2.4.3. Serum Chemistry Analysis

Approximately 0.5 mL of whole blood samples from each rat was placed into EP tubes and left to stand for 2 h to separate serum from blood. The obtained sera samples were analyzed by a clinical chemistry analyzer (Hitachi 7020). And this analysis of our study contained the following indexes: albumin (ALB), alkaline phosphate (ALP), alanine aminotransferase (ALT), aspartate aminotransferase (AST), total protein (TP), total bilirubin(TBIL), glucose (GLU), blood urea nitrogen (BUN), and creatinine (CREA).

#### 2.4.4. Statistical Analyses

Refering to the similar method adopted by the previous report [31], data obtained from our tests were analyzed by using SPSS software. All quantitative data were expressed as “mean value ± standard deviation”, and a value of *P* ≤ 0.05 was considered as significantly different.

### 2.5. Histopathologic Study

Sixty mice were equally divided into five time groups of 2 h, 4 h, 24 h, 3 days, and 7 days and a control group (*n* = 10, 5 male and 5 female mice). After being fasted over night, animals of five time groups were given P(ECA-IA-MEG) hydrogel suspension by gavage method at a dose of 7500 mg/kg body weight, in the volume of 0.3 mL/10 g body weight, and the control group was given a same volume of 1% CMC-Na solution.

Then, at time points of 2 h, 4 h, 1 day, 3 days, and 7 days after administration, one corresponding group of mice were sacrificed and the following organs were extracted: heart, liver, spleen, lung, kidney, stomach, intestine (duodenum, jejunum, ileum, and colon), and testes. The obtained tissue samples were fixed via preserving in 10% buffered formaldehyde for 48 h, and then embedded in paraffin, sectioned at 5 *μ*m, and visualized by hematoxylin and eosin staining [32].

## 3. Results and Discussion

### 3.1. Synthesis and Characterization of Macromonomer and Hydrogel

#### 3.1.1. Synthesis of P(ECA-IA-MEG) Hydrogel

**Figure 1:**
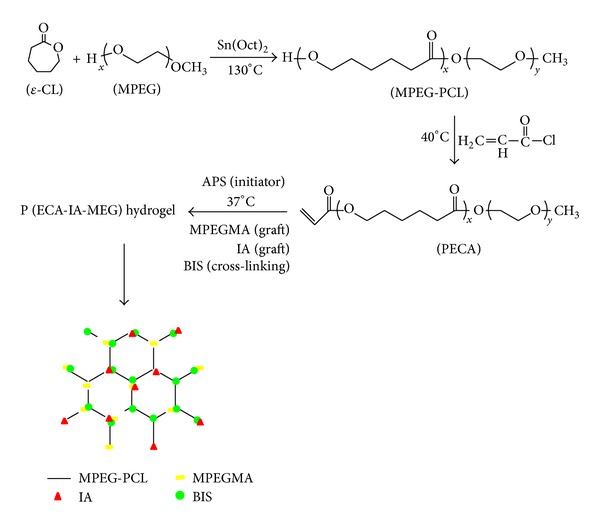
Synthesis scheme of P(ECA-IA-MEG) hydrogel.

**Figure 2:**
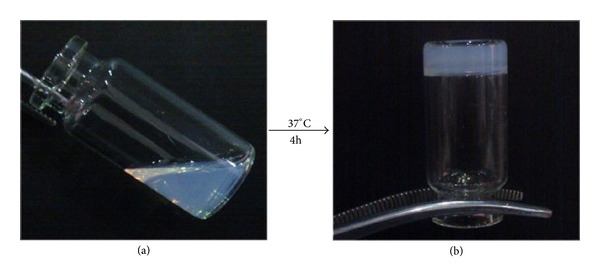
Direct observation of synthesis of the P(ECA-IA-MEG) hydrogel. (a) Mixture solution of PECA, IA, MPEGMA, BIS, and APS. (b) Obtained hydrogel after being heated at 37°C for 4 hrs.

The MPEG-PCL copolymer was synthesized by ring-opening polymerization of *ε*-caprolactone initiated by MPEG. In our previous work, the PECA copolymer was proved to be a biocompatible material with low cytotoxicity. It showed the synthesis scheme of PECA macromonomer from Figure 1. As also shown in Figure 2, the sample had good solubility in water and could form clear hydrogel after being soaked and bathed in water at 37°C for 4 hours.

#### 3.1.2. ^1^H-Nuclear Magnetic Resonance (^1^H-NMR) of the PECA

**Figure 3:**
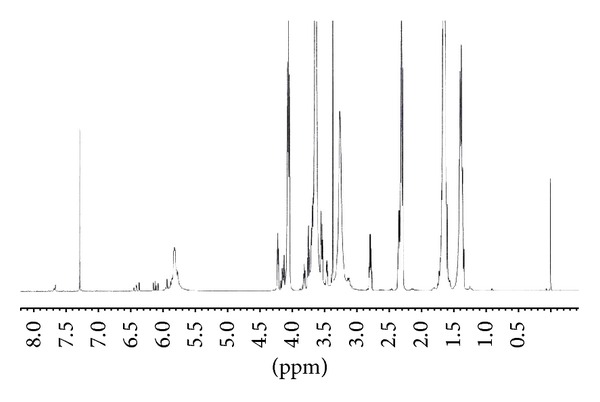
^1^H NMR spectrum of PECA macromonomer (in CDCl_3_).

Figure 3 presented the ^1^H NMR spectrum of PECA macromonomer. The peaks at 1.3, 1.6, 2.3, and 4.1 ppm pertained to methane protons (–CH_2_–) of PCL blocks. The signals at 3.3 ppm were attributed to the methyl protons (–CH_3_) of MPEG blocks. The methane protons (–CH_2_–) of MPEG segment appeared at 3.7, 4.1 ppm, while the peaks at 5.8, 6.1, and 6.4 ppm belong to protons of the C=C. The results demonstrated that MPEG-PCL copolymer was successfully prepared and reacted with acryloyl chloride.

#### 3.1.3. Fourier Transform Infrared (FT-IR) of Macromonomer and Hydrogel

**Figure 4:**
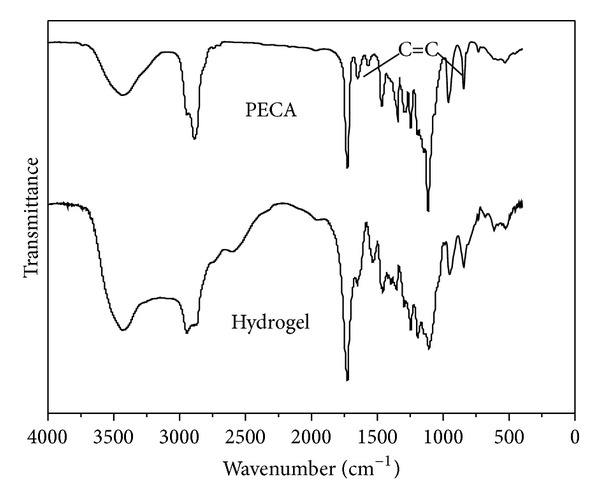
FT-IR spectra of PECA and P(ECA-IA-MEG) hydrogels.

P(ECA-IA-MEG) hydrogel was synthesized by heat-initiated free radical polymerization.  Figure 4 showed the FT-IR spectra for PECA macromonomer and P(ECA-IA-MEG) hydrogel. In Figure 4, the absorption bands at 1690.3 cm^−1^ and 1115.7 cm^−1^ were assigned to ester and ether of PCE stretching vibration modes, respectively. The absorption bands at 1637 cm^−1^ and 835.6 cm^−1^ were attributed to C=C stretching of PECA macromonomer. But they decreased greatly in the FT-IR of P(ECA-IA-MEG) hydrogel, which indicated that the end double bonds had been converted to carbon-carbon single bonds completely during the formation of hydrogel. It could be seen that the P(ECA-IA-MEG) hydrogel was successfully prepared.

#### 3.1.4. Morphological Characterization of the P(ECA-IA-MEG) Hydrogel

**Figure 5:**
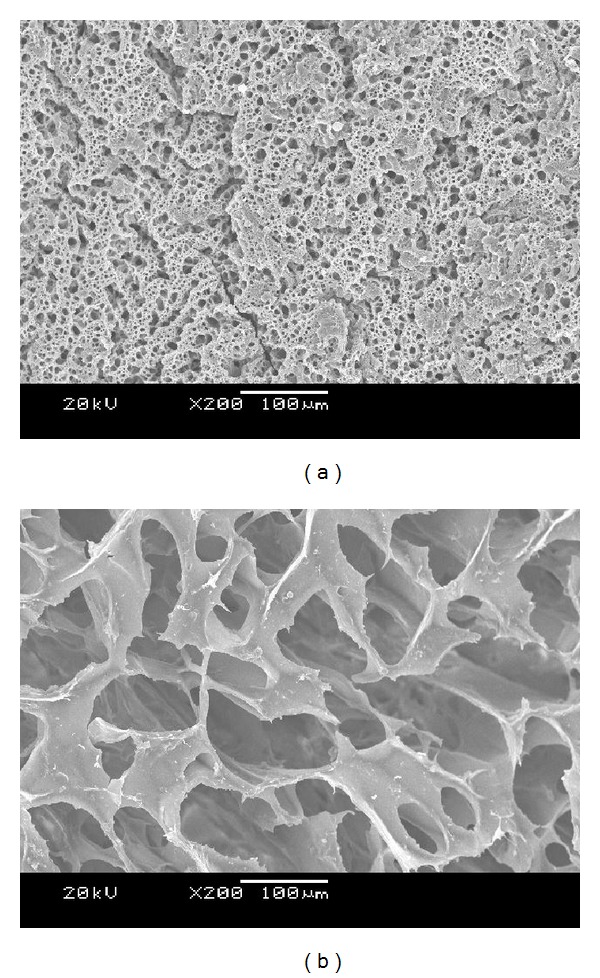
SEM observation of P(ECA-IA-MEG) in pH 1.2 (a) and pH 6.8 (b).

SEM was used to observe the cross section of P(ECA-IA-MEG) hydrogel in different aqueous media with pH 1.2 and 6.8. As clearly seen in Figure 5, there were many pores in the hydrogels and the pores were interconnected with each other. In pH 1.2, most carboxylic acid groups were in the form of COOH, and they formed the hydrogen bonds with the section of MPEG. The hydrogels were shrinking, and the mesh sizes were small. Conversely, when environmental pH value increased to 6.8, the hydrogen bond broke owing to the ionization of carboxylic acid groups, meanwhile, electrostatic repulsion caused the network to expand. The hydrogels swelled obviously and the mesh size increased a lot. These figures implied the pH-sensitive characterization of the prepared hydrogel, and indicated that the hydrogels have a good pH-responsibility.

#### 3.1.5. Swelling Behavior of the Hydrogels

In order to investigate the influence of the external pH value on the water absorption behavior of obtained hydrogels, the hydrogels were immersed in different pH conditions (pH 1.2 and pH 7.4), and the results were showed in Table 2. It was seen that the equilibrium swelling ratios of hydrogels in pH 7.4 buffer solutions were much higher than those in pH 1.2 buffer solutions. Besides, the swelling ratios in pH 7.4 buffer solutions became higher with time, while they became lower in pH 1.2 buffer solutions. In pH 1.2, the hydrogels were shrinking because of the hydrogen bonds, and the water was squeezed out from the hydrogels, while in pH 7.4, the hydrogen bonds broke and the electrostatic repulsion made the hydrogels swelling. The water entered the hydrogels easily, and the swelling ratios were increased rapidly. These data showed that the environmental pH value had a great influence on the swelling behavior of hydrogels and that the hydrogels were pH-sensitive.

### 3.2. Acute Oral Toxicity Test

#### 3.2.1. General Conditions

Clinical manifestation observed in acute oral toxicity test was listed in Table 3. Neither poisoning performance nor toxic response was demonstrated during the seven-day observation period, and all mice had behaved normally. To elaborate, they were sensitive to sound, light, and other stimuli. Their furs remained totally normal without any ulceration. It was noticeable that no running nose, eye secretion, salivation, or vomit were observed, and no mouth or nose dryness or edema were showed in this treatment group. Besides, the mice's feces were in regular form with normal color without mucus, pus, or blood. Then After necropsy, no macroscopic pathological alterations caused by P(ECA-IA-MEG) hydrogel were found in all mice. All above lines of evidence showed that P(ECA-IA-MEG) hydrogel was safe for taking orally.

#### 3.2.2. Body Weight of Mice

**Figure 6:**
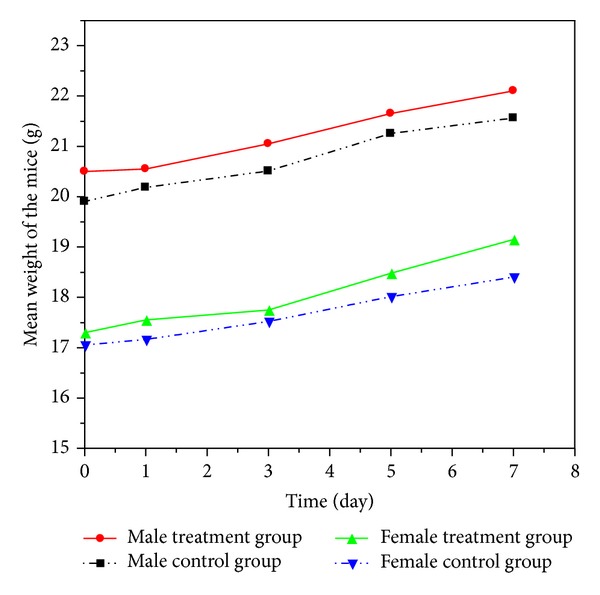
Mice body weight of each group during the observation period.

Both the control and treatment groups were weighted and recorded accordingly during the whole seven-day period. As illustrated by the body weight curves in Figure 6, no significant difference was showed between the treatment and control group; thus, P(ECA-IA-MEG) hydrogel would not affect the body weight of the mice.

#### 3.2.3. Maximal Tolerance Dose

Due to P(ECA-IA-MEG) hydrogel's excellent biocompatibility, no lethal dose or median lethal dose could be obtained; thus, MTD method was used as an alternative to evaluate the acute toxicity of P(ECA-IA-MEG) hydrogel. Since mortality rates remain zero after being treated with highest tested dose, MTD of P(ECA-IA-MEG) hydrogel was higher than 10000 mg/kg body weight by oral administration for BALB/c mice. Higher dose could cause side effects and possibly lead to death. This is due to the limited gastric capacity of mice rather than the toxicity of the hydrogel. In order to ensure that all toxicity was caused by P(ECA-IA-MEG) hydrogel instead of excessive volume, dose was designed at 7500 mg/kg for the rest of the experiments.

#### 3.2.4. Hematology Routine Test and Serum Chemistry Analysis

The purpose of hematology routine test was to investigate whether the P(ECA-IA-MEG) hydrogel could lead to the blood system's abnormality. From the results listed in Table 4, we could find that there were no important adverse effects in the hematology variables both in male or female treatment group. Some statistically significant differences from control (*P* ≤ 0.05) were observed in white blood cell count (female treatment group, 74% of control) and in hematocrit (male treatment group, 107% of control). However, these difference were not considered to be adverse because the differences were relatively minor in magnitude (26% lower WBC and 7% higher HCT, resp.).

The serum chemistry analysis was designed to check the liver functions (ALB, ALP, ALT, AST, TBIL, and TP), renal functions (BUN and CREA), and blood sugar level (GLU). In Table 5, compared with the control group, no important biologically adverse effects were observed in all the listed clinical chemistry parameters in both male and treatment groups. Additionally, statistically significant increases (*P* ≤ 0.05) were observed in blood urea nitrogen and creatinine in the female treatment (111% and 125% of control group, resp.). These differences were also relatively small in magnitude (11% higher in BUN and 25% higher in CREA).

Both of these results indicated that P(ECA-IA-MEG) hydrogel did not affect the bloody system, liver function, and renal function of mice. However, it was difficult to eliminate the fluctuations between treatment group and control group, because the individual differences existed inevitably.

### 3.3. Histopathologic Study

No symptoms of poisoning were identified after giving P(ECA-IA-MEG) hydrogel by gavage at a dose of 7500 mg/kg body weight. In order to trace the toxicity caused by P(ECA-IA-MEG) hydrogel accurately, histopathologic changes of major organs were checked in a sequential order of 2 h, 4 h, 1 day, 3 days, and 7 days, respectively.

All samples of major organs obtained were observed by light microscope, but no obvious histopathological lesions were found. Since P(ECA-IA-MEG) hydrogels should have been metabolized completely, pictures for each organ at the time point of 7th day were demonstrated in this paper.

**Figure 7:**
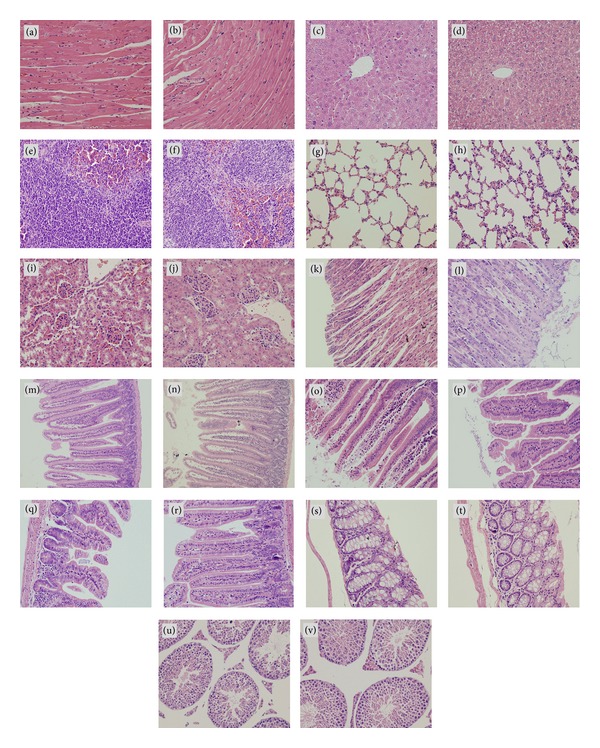
Photograph of mice cardiac muscle, liver, spleen, lung, kidney, stomach, duodenum, jejunum, ileum, colon, and testes after oral administration of P(ECA-IA-MEG) hydrogel (40x), ((b), (d), (f), (h), (j), (l), (n), (p), (r), (t), (v)) and of the control group ((a), (c), (e), (g), (i), (k), (m), (o), (q), (s), (u)).

Figures 7(a) and 7(b) exhibited the comparison between the light microscopic images of cardiac muscle of treatment group and control group on the 7th day. Cardiac myocytes displayed orderly and clearly, without any inflammatory exudate, necrosis, or hemorrhage.

Figures 7(c) and 7(d) presented difference between the optical micrograph of livers of animals treated with and without P(ECA-IA-MEG) hydrogels on the 7th day. Under the microscope, no obvious degeneration and necrosis were found. The dividing lines of liver lobules were clear and the hepatic cord arranged in neat order. No hypertrophy and hyperemia were observed on hepatic sinusoid, and no neutrophil, lymphocyte, or macrophage infiltration was found.

As shown in Figures 7(e) and 7(f), there was little difference between the hydrogel-treated lung and the control group. Additionally, the tissue structure of hydrogel-treated lung did not show any bronchioles, alveoli ectasia, or collapse, meanwhile, no inflammatory cell infiltration surrounding the bronchus was identified.

The structure of spleen for both groups was represented In Figures 7(g) and 7(h). No pathologic changes were showed; thus, the spleen sinus was absolutely normal, without showing any pathologic changes.

Figures 7(i) and 7(j) exhibited the cytopathologic photos of mice kidney. The treated kidney was in ordinary shape compared with the control one. No degeneration, bleeding, necrosis was showed within renal glomerulus and various kidney tubes.

The cytopathologic photographs of gastrointestinal tract were also observed (Figures 7(k)–7(t)). The gastrointestinal tract included stomach, duodenum, jejunum, ileum, and colon. From these photographs, it was easy to find that gastric glands and intestinal glands were in regular arrangement, mucosa cells were clear, and the basement membranes were intact. Haemorrhage, hydropsy, inflammatory cell infiltration, degeneration, and necrosis were largely absent in the hydrogel-treated experimental group as well as the control group.

Figures 7(u) and 7(v) described the light micrograph of male mice testis. Histopathologic examination of the spermary showed no significant pathologic changes.

## 4. Conclusion

In this paper, a kind of pH-sensitive P(ECA-IA-MEG) hydrogel was successfully prepared by heat-initiated free radical polymerization method. The chemical and physical characterization reflected that the hydrogel was pH-sensitive. The results of acute oral toxicity evaluation showed that there was no toxic response or histopathological changes caused by P(ECA-IA-MEG) hydrogel in BALB/c mice by gastric perfusion. Thus, the hydrogel prepared in this paper might be a safe candidate for application in biomedical field, especially in oral drug delivery system.

## Figures and Tables

**Table 1:** The hydrogel prepared in this work.

PECA : MPEGMA : IA	BIS content (wt%)	IA Content (wt%)
3 : 3 : 4	8.3	16.6

**Table 2:** Swelling behavior of hydrogels in aqueous medium with pH 1.2 and pH 7.4 at 37°C.

Time (h)	Swelling Radio (%)	
	pH 1.2	pH 7.4
1	210 ± 3	341 ± 16
2	205 ± 10	355 ± 20
4	204 ± 6	383 ± 26
8	202 ± 5	402 ± 11
24	200 ± 3	426 ± 14
48	195 ± 4	460 ± 12
72	192 ± 3	480 ± 15

**Table 3:** Toxicity signs not observed in BALB/c mice treated with P(ECA-IA-MEG) hydrogel.

Motor activities	Restlessness, hyperactivity, and phonation
Symptoms of nervous system	Tail erect, tremble, spasm, movement disorder, and gesture abnormal
Symptoms of autonomic Nervous system	Protrusion of eyeballs, salivation, weep, urination, diarrhea, hair bristled up, and discoloration, dyspnea
mortality	Death

**Table 4:** Hematology routine test of acute oral toxicity test.

	Female control group	Female treatment group	Male control group	Male treatment group
WBC (10^9^/L)	6.01 ± 1.10	4.45 ± 0.59*	3.86 ± 0.69	4.06 ± 0.50
RBC (10^12^/L)	11.13 ± 0.51	11.36 ± 0.39	11.11 ± 0.37	11.33 ± 0.38
HGB (g/L)	149.57 ± 5.47	152.63 ± 3.29	145.57 ± 3.95	151.50 ± 6.00
HCT (%)	58.17 ± 0.89	59.85 ± 1.53	57.30 ± 0.88	61.25 ± 1.77*
PLT (10^9^/L)	537.43 ± 108.23	666.38 ± 121.95	635.86 ± 56.57	666.38 ± 48.92
MCV (fL)	51.77 ± 1.18	52.33 ± 0.93	52.79 ± 0.90	53.84 ± 0.80
MCH (pg)	13.37 ± 0.31	13.36 ± 0.39	13.09 ± 0.38	13.31 ± 0.25
MCHC (g/L)	258.14 ± 3.72	255.38 ± 6.12	247.71 ± 4.92	247.38 ± 4.37
RDW (%CV)	16.89 ± 2.98	17.34 ± 0.92	17.31 ± 0.80	17.53 ± 0.70
MPV (fL)	5.53 ± 0.68	5.80 ± 0.55	5.39 ± 0.47	5.70 ± 0.25
PDW (%)	13.54 ± 0.81	14.77 ± 0.81	14.05 ± 0.79	15.06 ± 0.87

*P ≤ 0.05 compared to control group.

**Table 5:** Serum chemistry analysis of acute oral toxicity test.

	Female control group	Female treatment group	Malecontrol group	Male treatment group
ALB	35.61 ± 1.57	35.00 ± 0.76	34.07 ± 1.35	34.34 ± 1.13
ALP	220.57 ± 16.24	236.38 ± 29.60	225.00 ± 29.30	206.00 ± 15.89
ALT	51.86 ± 18.77	43.75 ± 6.23	42.43 ± 3.21	42.13 ± 4.58
AST	113 ± 14.60	109.38 ± 12.64	102.00 ± 26.88	100.88 ± 19.11
BUN	9.01 ± 0.55	10.02 ± 1.10*	9.89 ± 1.47	9.45 ± 0.99
CREA	1.86 ± 1.46	2.33 ± 1.06*	2.43 ± 1.51	2.90 ± 0.76
GLU	3.61 ± 0.56	3.98 ± 0.54	5.37 ± 1.34	5.32 ± 0.88
TBIL	0.07 ± 0.19	0.27 ± 0.24	0.37 ± 0.33	0.21 ± 0.22
TP	62.54 ± 3.37	60.78 ± 1.89	60.31 ± 2.46	61.44 ± 2.00

*P ≤ 0.05 compared to control group.
